# Supplementary material for: Setting new boundaries of 16S rRNA gene identity for prokaryotic taxonomy
Source: Int J Syst Evol Microbiol. 2025 Apr 7;75(4):006747. doi: 10.1099/ijsem.0.006747 (PMC12281934; doi:10.1099/ijsem.0.006747)
Supplement: Uncited Supplementary Material 1. [file ijsem-75-06747-s001.pdf]

Supplementary Material for

Setting new boundaries of 16S rRNA gene identity for prokaryotic taxonomy

Timothy J. Hackmann*

*Correspondence: Timothy J. Hackmann, tjhackmann@ucdavis.edu

This PDF file includes:

Fig. S1 to S6
Legends for Data S1 to S2

Other Supplemental Material for this manuscript includes the following:

Data S1 and S2

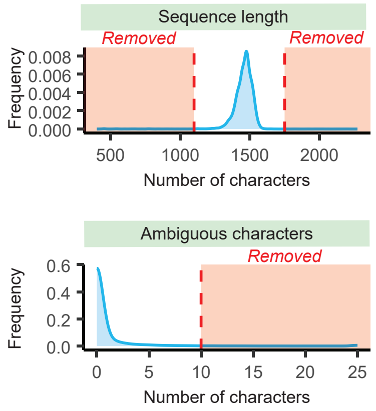

Fig. S1. Ribosomal sequences vary in length and number of ambiguous characters, and the most extreme were removed from our analysis.

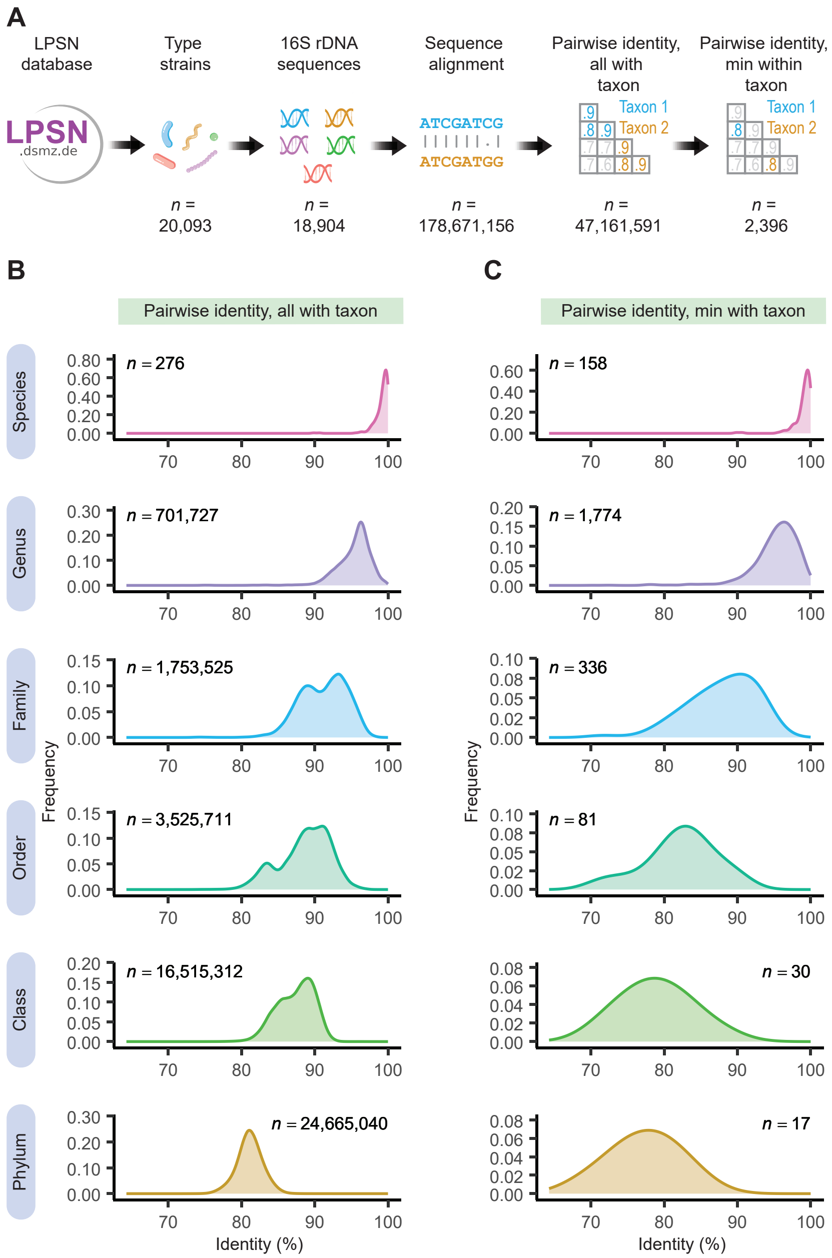

D

	All within taxon (%)			Min within taxon (%)			Bounds for classification (%)	
Rank	Median	P5	P95	Median	P5	P95	Current	Literature
Species	99.5	97.7	100	99.4	97.2	100	97.2 to 100	98.7 to 100
Genus	95.8	91.4	98.4	95.8	90.1	99.0	90.1 to 99.0	94.5 to <98.7
Family	91.5	86.0	95.9	88.6	79.7	94.0	79.7 to 94.0	86.5 to <94.5
Order	89.2	82.3	93.5	82.9	72.7	89.7	72.7 to 89.7	82.0 to <86.5
Class	87.7	83.0	91.0	78.7	72.1	86.9	72.1 to 86.9	78.5 to <82.0
Phylum	81.1	78.1	84.1	77.5	69.7	83.8	69.7 to 83.8	75.0 to <78.5

[1]P5 = 5th percentile
[2]P95 = 95th percentile

Fig. S2. Sequence identity varies within taxonomic ranks of bacteria. Same as Fig. 1, but with bacteria alone.

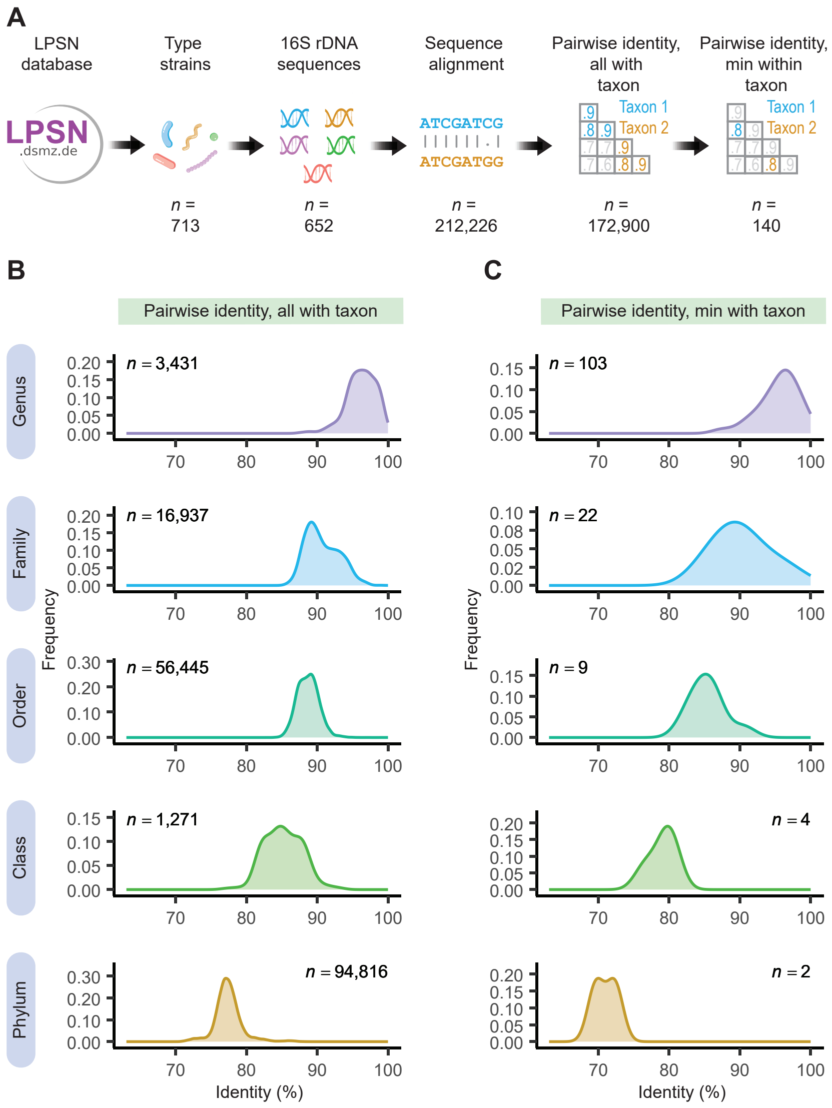

D

Rank	All within taxon (%) Median	P5	P95	Min within taxon (%) Median	P5	P95	Bounds for classification (%) Current	Literature
Genus	96.3	92.4	99.1	96.1	90.6	99.1	90.6 to 99.1	94.5 to <98.7
Family	90.3	87.4	94.8	89.6	85.1	96.7	85.1 to 96.7	86.5 to <94.5
Order	88.7	86.3	91.2	85.3	82.6	88.9	82.6 to 88.9	82.0 to <86.5
Class	85.1	81.4	89.2	79.6	76.9	80.3	76.9 to 80.3	78.5 to <82.0
Phylum	77.3	74.7	80.6	71.0	69.8	72.2	69.8 to 72.2	75.0 to <78.5

[1]P5 = 5th percentile
[2]P95 = 95th percentile

Fig. S3. Sequence identity varies within taxonomic ranks of archaea. Same as Fig. 1, but with archaea alone.

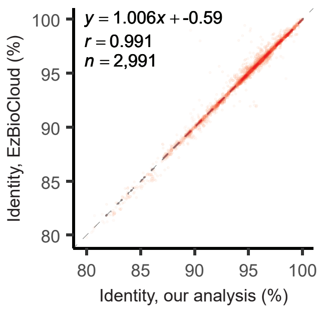

Fig. S4. Pairwise identity calculated in our analysis closely matches that of EzBioCloud.

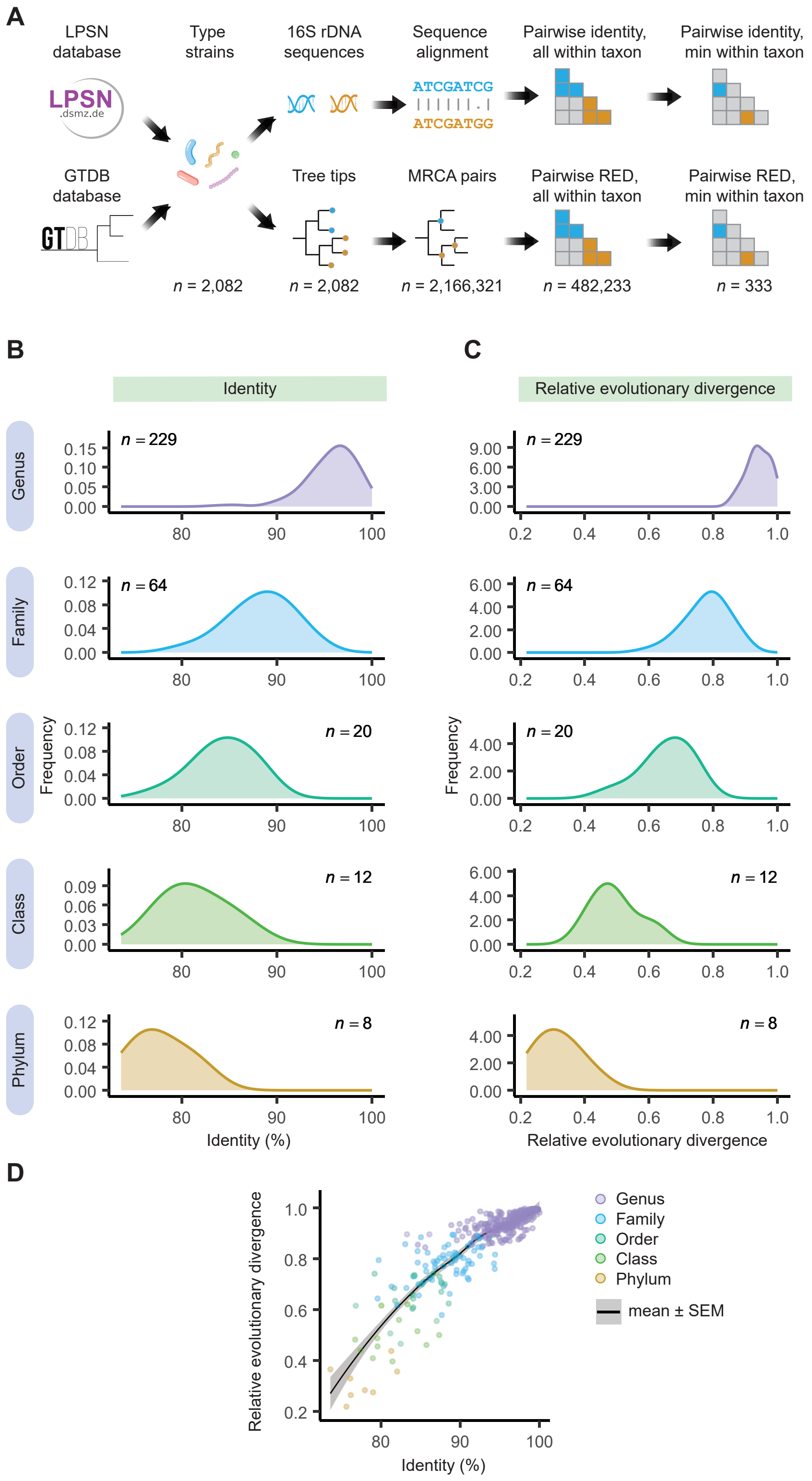

Fig. S5. Sequence identity is correlated with relative evolutionary divergence (RED) in bacteria. Same as Fig. 2, but with bacteria alone.

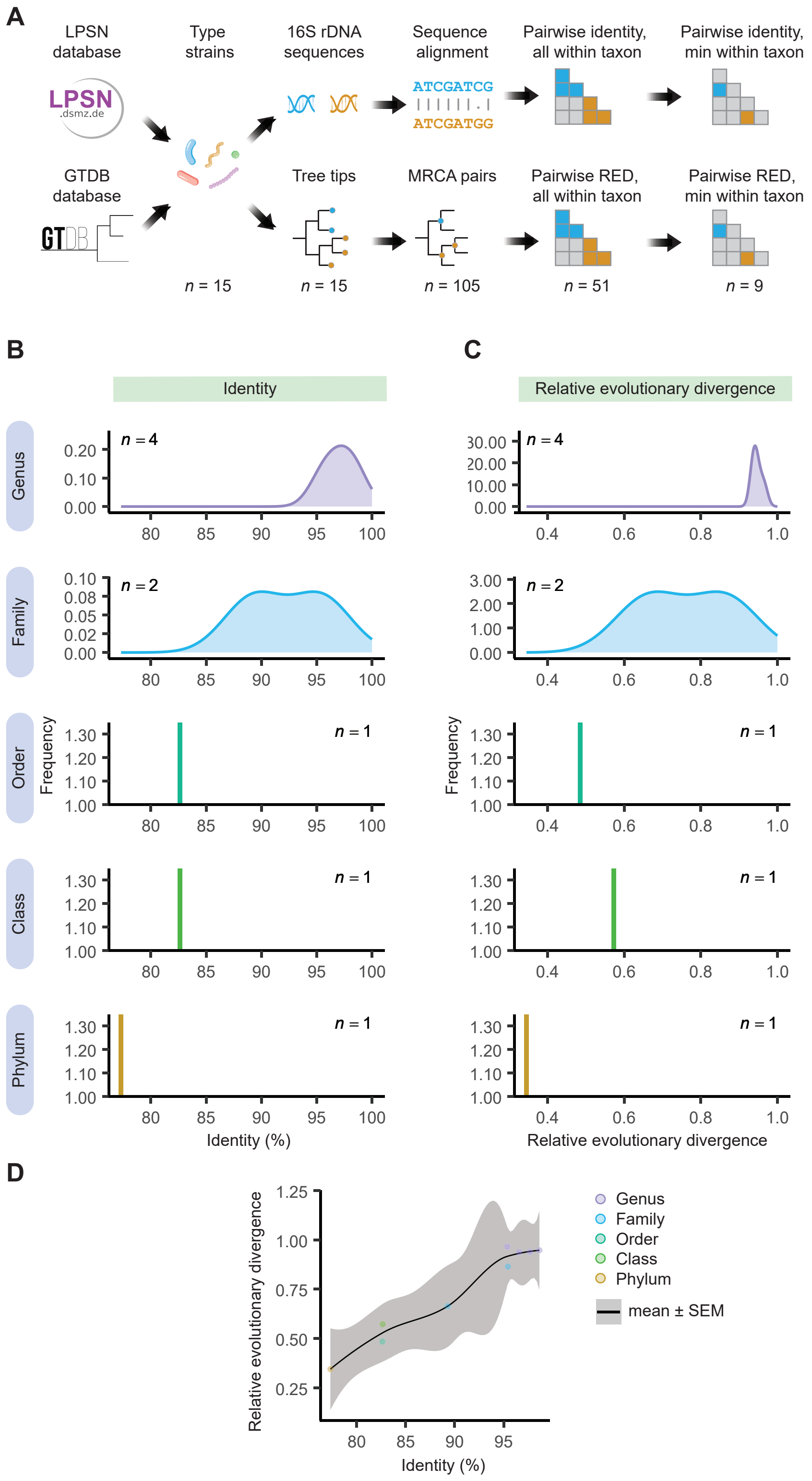

Fig. S6. Sequence identity is correlated with relative evolutionary divergence (RED) in archaea. Same as Fig. 2, but with archaea alone.

Data S1. (separate file)
Taxonomy and sequences of strains used in our analysis.

Data S2. (separate file)
Values pairwise identity summarized by taxon.
